# Estimating shifts in diversification rates based on higher-level phylogenies

**DOI:** 10.1098/rsbl.2016.0273

**Published:** 2016-10

**Authors:** Tanja Stadler, Jana Smrckova

**Affiliations:** 1 Department of Biosystems Science and Engineering, ETH Zürich, 4058 Basel, Switzerland; 2 Swiss Institute of Bioinformatics (SIB), 1015 Lausanne, Switzerland; 3 Department of Zoology, Faculty of Science, University of South Bohemia, 37005 Ceske Budejovice, Czech Republic

**Keywords:** phylogenetic tree, inference, speciation, extinction, incomplete sampling

## Abstract

Macroevolutionary studies recently shifted from only reconstructing the past state, i.e. the species phylogeny, to also infer the past speciation and extinction dynamics that gave rise to the phylogeny. Methods for estimating diversification dynamics are sensitive towards incomplete species sampling. We introduce a method to estimate time-dependent diversification rates from phylogenies where clades of a particular age are represented by only one sampled species. A popular example of this type of data is phylogenies on the genus- or family-level, i.e. phylogenies where one species per genus or family is included. We conduct a simulation study to validate our method in a maximum-likelihood framework. Further, this method has already been introduced into the Bayesian package MrBayes, which led to new insights into the evolution of Hymenoptera.

## 1. Introduction

A key goal in macroevolution is to identify changes in the rates of diversification and to find causal explanations for variations in the species diversity we observe today. It has been shown that species phylogenies based on only extant taxa and no extinct lineages can be used to infer both the speciation and extinction rates, and thus in particular the diversification rate defined as speciation rate minus extinction rate [1–3].

Many organismal clades contain a vast amount of species, which makes construction of complete species phylogenies an arduous task. Although for some species groups complete or near-complete phylogenies have been already inferred [4,5], many others are still available as phylogenies on a higher taxonomic level only, meaning that only one species per higher taxonomic unit such as genus or family is included [6–10]. We call such phylogenies ‘higher-level phylogenies’.

Computational methods were developed to estimate diversification rates from higher-level phylogenies [7,11–14]. While Paradis [11] and Stadler & Bokma [14] have devised a method for estimating constant speciation and extinction rates in higher-level phylogenies, Rabosky *et al*. [12], Alfaro *et al*. [7] and Rabosky [15] refined these approaches by also allowing for the computation of speciation and extinction rate variation across clades. Additionally, rate variation through time may be induced by external variables, such as climate, break-up of continents, sea-level changes or development of key innovations or competition. In this paper, we introduce a framework allowing for the estimation of changes in diversification rates through time from higher-level phylogenies. Our mathematical equations derived here have been implemented into MrBayes, and used to infer a phylogeny of Hymenoptera through a Bayesian approach [16].

In what follows, we present a maximum-likelihood method to estimate changes in diversification (= speciation − extinction) rates and turnover (= extinction/speciation) for *higher-level phylogenies* where all phylogenetic relationships are resolved up to a certain point in time, and each clade, descending a lineage at that point in time, is collapsed to one tip. We show in a simulation approach that shifts in diversification rates can be estimated reliably based on our likelihood framework. We then explain how we can transform a phylogeny on the genus- or family-level into a higher-level phylogeny to analyse empirical data.

## 2. Methods

### (a) Birth–death–skyline model

We extend the *constant rates birth–death process* (crBDP; [17–19]), to the *birth–death–skyline process*, following Stadler [20] and Stadler *et al*. [21].

The crBDP starts with a single lineage at time *x*_0_ in the past (stem age) and gives birth to descendant lineages with a constant rate of speciation *λ* and lineages die with a constant rate of extinction *μ*. At the present time, the process is stopped. The *reconstructed phylogenetic tree* is acquired by pruning all lineages that went extinct.

The birth–death–skyline process generalizes the crBDP by allowing for rate changes through time: time between the present and *x*_0_ is split up through 0 = *t*_0_ < *t*_1_ < *t*_2_ < … < *t_m_* < *x*_0_. The present day is depicted as *t*_0_*.* Speciation and extinction rates are constant (*λ_i_* and *μ_i_*) between *t_i_* and *t_i_*_+ 1_, and may differ arbitrarily between intervals. For estimating the parameters of the birth–death–skyline process, the probability of a reconstructed phylogeny given the parameters is provided in Stadler [20].

### (b) Higher-level trees

A higher-level phylogeny is obtained from a complete species phylogeny by pruning the extant descendants of every lineage at time *x*_cut_ in the past to one sampled lineage together with the information on the number species represented by each sampled lineage (see e.g. fig. 1*d* in [14]). Let the branching times in the phylogeny be *x*_1_ > *x*_2_ … > *x_n_*_− 1_, and let the number of species represented by tip *i* be *n_i_*. We derived the probability of a higher-level phylogeny, in order to estimate maximum-likelihood diversification rates and turnover.

### (c) Simulations

We investigate the accuracy of parameter estimation based on simulated higher-level phylogenies. First, we simulated species trees with 2000 tips under different diversification scenarios with one rate shift and disparate rates of speciation (resp. extinction) before and after the shift using the R package TreeSim [22]. We then collapsed clades to obtain higher-level phylogenies by pruning each clade at time *x*_cut_ before the present to one lineage terminating at the present with the information of number of species in the clade [23,24]. We have chosen times of *x*_cut_ corresponding to three quartiles of the age of the tree, i.e. 25%, 50% and 75%.

In the simulated trees, *λ*_y_ (y for young) denotes the speciation rate between the rate shift time and the present, and *λ*_o_ (o for old) is the speciation rate ancestral to the rate shift time. We set parameters specifying decelerating and accelerating diversification, namely birth rates were *λ*_y_ = 0.5*, λ*_o_ = 1 and *λ*_y_ = 1, λ_o_ = 0.5 for trees with decreasing and increasing diversification rate, respectively. Death rate was held constant with *μ* = 0.1 in these simulations. For both the accelerating and decelerating scenario, we run simulations with both a time of the shift at 2 myr before present (BP) and 3.5 myr BP. Second, we increased the extinction rate to *μ* = 0.4 (and thus increased turnover) under decreasing diversification (*λ*_y_ = 0.5, *λ*_o_ = 1). For this setting, which induces a recent diversification rate of 0.1, we chose a rate shift at 8 Mya. This setting is comparable to the simulation setting with a rate shift at 2 Mya and a recent diversification rate of 0.4, as both settings induce a lineage accumulation between the rate shift at the present of 0.8 (= 8 × 0.1 = 2 × 0.4); thus, these settings induce roughly the same number of lineages at the time of the rate shift. Last, we fixed the speciation rate to *λ* = 1.0 under decreasing diversification (*μ*_y_ = 0.6*, μ*_o_ = 0.1). We chose a rate shift time of 2 Mya, again ensuring a lineage accumulation since the rate shift of 0.8 (= 2 × 0.4). Thus, we have six different settings. For each set of parameters, 100 trees were produced.

For all trees, maximum-likelihood estimates of diversification rate *λ* − *μ* and turnover *μ*/*λ* before and after the rate shift, together with the time of the rate shifts, have been obtained using TreePar v. 3.3 function bd.shifts.optim [25] with the ‘groups’ option. This function employs equation (3.1) below.

The code required to perform our analyses is provided in the electronic supplementary material.

## 3. Results

### (a) Probability of a higher-level phylogeny

The probability density of a higher-level phylogeny *𝒯* with *n* tips is derived in the electronic supplementary material, theorem 3,3.1
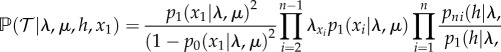
where *p_k_*(*t*|*λ*,*μ*) is the probability that a lineage at time *t* in the past has *k* descendants at present time 0, and 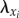 is the speciation rate at time *x_i_*. This is equivalent to (appendix, theorem 1)
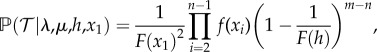
with, for *t* in (*t_i_*, *t_i_*_+ 1_], and re-defining *t_i_*_+ 1_ := *t* for convenient notation
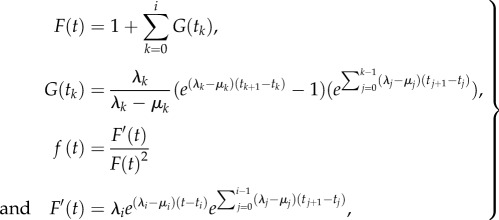
i.e. we do not need to specify how many species belong to each tip, but we need to know only the total number of species *m*. In fact, even if we knew how many species belong to each tip, that would not improve parameter estimates.

### (b) Diversification rate estimates

**Figure 1.**
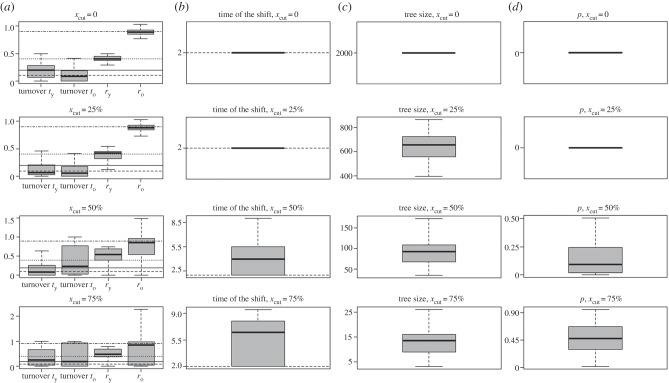
Results of a simulation study for trees with constant extinction rate (0.1) and decreasing diversification rate (from 0.9 to 0.4) with a rate shift at 2 myr BP. Central tendency in boxplots is median, vertical lines indicate original values used in simulations. Rows show results for trees with increasing *x*_cut_. Plots in column (*a*) depict the estimated maximum-likelihood turnover and diversification rate parameters. Parameters between the present and the time of the shift and before the shift are denoted as *t*_*n*_, *r*_y_, and *t*_*o*_ and *r*_o_, respectively. Plots in column (*b*) estimated maximum-likelihood shift times in myr, (*c*) sizes of the analysed trees and (*d*) *p*-values of the likelihood ratio test (using the Chi-squared distribution with 3 degrees of freedom, for speciation rate, extinction rate and shift time) comparing a single rate shift with a constant rate model.

**Figure 2.**
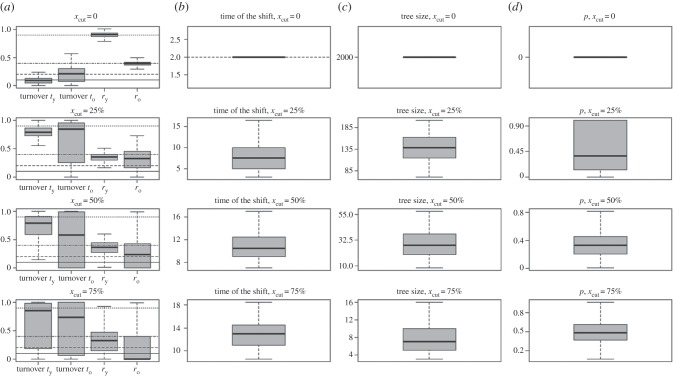
Results of a simulation study for trees with constant extinction rate (0.1) and increasing diversification rate (0.4–0.9) with a rate shift at 2 myr before present.

In all simulations, diversification rates, turnover and times of the shifts were estimated correctly when analysing fully sampled species-level phylogenies (figures 1 and 2; electronic supplementary material, figure S1–S4). With the complete trees, and *x*_cut_ at 25% with decreasing diversification and constant extinction rate, we consistently identified one significant rate shift. With increasing *x*_cut_, we lost significance, and the times of the (potentially not significant) shifts were estimated to be older than initially simulated.

The better performance of constant speciation rate (figure 1) versus constant extinction rate (electronic supplementary material, figure S4) under the same diversification rates is most likely owing to the latter having a higher turnover, thus a more pronounced pull-of-the-past, and thus fewer lineages at the time of the rate shift causing less strong signal.

Simulation, in general, showed much better precision of estimates retrieved for incomplete trees with decelerating diversification than for accelerating scenario. In phylogenies with diversification rate slowing down towards the present, precise values were retrieved for *x*_cut_ up to 50% of tree age, whereas for trees with accelerating evolutionary rate, reliable estimates have been obtained for complete species-level phylogenies only. This result makes intuitive sense: most branching events in accelerating trees occur close to tips of the tree, i.e. most of the branching events are removed even for small *x*_cut_, hence providing limited information for maximum-likelihood estimation.

## 4. Discussion

Not accounting for the sampling of higher-level taxa can lead to severe biases in parameter estimation, in particular an underestimation of extinction rate and turnover (see fig. 2 in [14]). We have formulated an inference framework for estimating shifts in diversification rate in higher-level phylogenies where all higher levels have the same age. Our simulations reveal that the method can estimate past diversification patterns from these higher-level phylogenies on extant species. Any phylogeny can be converted into a higher-level phylogeny by collapsing all clades descending from a lineage at time *x*_cut_*,* where *x*_cut_ is a time point prior to which the phylogeny is fully resolved.

It has been shown that incorporating fossils will dramatically improve the quality of diversification rate estimates: in particular, the extinction rate can be estimated far better [26]. While Silvestro *et al*. [27] applied the model presented here to only fossil data, Zhang *et al*. [16] combined equation (3.1) of this paper with a fossilization process. Thus, coherent analysis of fossils and extant species data became possible. The resulting so-called fossilized birth–death process [28] has been implemented into MrBayes and a higher-level phylogeny with fossils of Hymenoptera has been inferred using the total-evidence-dating. The analyses of Zhang *et al*. [16] revealed that not accounting for the higher-level phylogeny structure, but assuming each species was sampled uniformly at random, has a drastic effect on tree inference. Thus, it is important to use appropriate diversification models not only for quantifying diversification rates, but also for inferring the phylogenies in the first place.

Here, we accounted for only one rate shift, even though our mathematical expression allows for an arbitrary number. The reason for this is that maximizing over multiple rate shift times is numerically very hard, and often leads the optimization tool to be stuck in local optima. In Stadler [20], a greedy approach for finding rate shifts was implemented, meaning the optimizer first searches for the best rate shift time, and with fixing this first rate shift time, it finds the second best rate shift time, etc. However, with the implementation of our mathematical equation into the Bayesian framework MrBayes, we can directly infer the posterior distribution of rate shifts and thus do not rely on a greedy approximation.

The birth–death–skyline process still makes a number of limiting assumptions, in particular that all species are assumed to be indistinguishable, hence all have the same speciation and extinction rates and the same probabilities of being sampled at any given time. Accordingly, a birth–death–skyline model cannot allow us to explicitly test scenarios of heritable rates [29] or clade-dependent rates (Medusa: [7], BAMM: [15]), although it can be used as a null model for testing more complex patterns of diversification. It remains a future challenge to combine complex models of rate variation across clades and through time, for inference of diversification rates based on higher-level phylogenies with fossils.

## Supplementary Material

Mathematical proofs

## Supplementary Material

Supplementary Figures

## Supplementary Material

R code
