# Supplementary material for: Estimating shifts in diversification rates based on higher-level phylogenies
Source: Biol Lett. 2016 Oct;12(10):20160273. doi: 10.1098/rsbl.2016.0273 (PMC5095187; doi:10.1098/rsbl.2016.0273)
Supplement: Mathematical proofs [file rsbl20160273supp1.pdf]

Biology Letters

Estimating shifts in diversification rates based on higher-level phylogenies

Supplementary information: mathematical proofs

Tanja Stadler & Jana Smrckova

Probability density of a strict higher-level phylogeny

Theorem 1. *The probability density of a strict higher-level oriented phylogeny under the birth-death-skyline plot with n sampled extant species representing m extant species is,*

$$\mathbb{P}(\mathcal{T}|\lambda,\mu,h,x_1) \;=\; \frac{1}{F(x_1)^2}\prod_{i=2}^{n-1} f(x_i)\left(1-\frac{1}{F(h)}\right)^{m-n}, \tag{1}$$

with, for t in $(t_i, t_{i+1}]$, and re-defining $t_{i+1} := t$ for convenient notation,

$$\begin{aligned}
F(t) &= 1+\sum_{k=0}^{i} G(t_k),\\
G(t_k) &= \frac{\lambda_k}{\lambda_k-\mu_k}\left(e^{(\lambda_k-\mu_k)(t_{k+1}-t_k)}-1\right)e^{\sum_{j=0}^{k-1}(\lambda_j-\mu_j)(t_{j+1}-t_j)},\\
f(t) &= F'(t)/F(t)^2,\\
F'(t) &= \lambda_i e^{(\lambda_i-\mu_i)(t-t_i)}e^{\sum_{j=0}^{i-1}(\lambda_j-\mu_j)(t_{j+1}-t_j)}.
\end{aligned}$$

Proof. Equation (1) directly follows from Eqn. (6) and (3) in [1], with $P(H<h)=1-\frac{1}{F(h)}$ given on page 117 of [1].

In order to determine $F(t)$, we use Proposition 5 in [1]. First note that we have to specify the stem age x_0 (denoted by T in [1]) to use Proposition 5. Let $x_0 > t$ be arbitrary. It will turn out that $F(t)$ does not depend on x_0. Proposition 5 yields, for t in $(t_i, t_{i+1}]$, and re-defining $t_{i+1} := t$ for convenient notation,

$$F(t) = 1+\sum_{k=0}^{i} G(t_k),$$

with

$$G(t_k) := \int_{t_k}^{t_{k+1}} \lambda(s) e^{\int_0^s (\lambda(u)-\mu(u))du} ds.$$

We note that compared to [1], we reverse time and thus also change the integration limits. We simplify,

$$\begin{aligned} G(t_k) &= \int_{t_k}^{t_{k+1}} \lambda_k e^{\int_0^s (\lambda(u)-\mu(u))du} ds \\ &= \int_{t_k}^{t_{k+1}} \lambda_k e^{(\lambda_k-\mu_k)(s-t_k)} e^{\sum_{j=0}^{k-1}(\lambda_j-\mu_j)(t_{j+1}-t_j)} ds \\ &= \frac{\lambda_k}{\lambda_k-\mu_k}\left(e^{(\lambda_k-\mu_k)(t_{k+1}-t_k)}-1\right) e^{\sum_{j=0}^{k-1}(\lambda_j-\mu_j)(t_{j+1}-t_j)}. \end{aligned}$$

The derivative $F'(t)$ equals the derivative of $G(t_i)$. □

Theorem 2. *Let $p_k(t|\lambda,\mu)$ be the probability that a lineage at time t in the past has k descendants at present time 0. Then, for $k>0$,*

$$p_k(t|\lambda,\mu) = (1-p_0(t|\lambda,\mu))\frac{1}{F(t)}\left(1-\frac{1}{F(t)}\right)^{k-1}.$$

Proof. The probability of a lineage present at time t before the present having $k \geq 1$ descendants at present conditioned on at least one descendant, is ([1], Equation 1),

$$\frac{1}{F(t)}\left(1-\frac{1}{F(t)}\right)^{k-1}.$$

Thus,

$$p_k(t|\lambda,\mu) = (1-p_0(t|\lambda,\mu))\frac{1}{F(t)}\left(1-\frac{1}{F(t)}\right)^{k-1}.$$

□

It remains to find an expression for $p_0(t|\lambda,\mu)$. This can be done in the following way.

Theorem 3. *An alternative way to express $P(\mathcal{T}|\lambda,\mu,h,x_1)$ is,*

$$\mathbb{P}(\mathcal{T}|\lambda,\mu,h,x_1) = \frac{p_1(x_1|\lambda,\mu)^2}{(1-p_0(x_1|\lambda,\mu))^2}\prod_{i=2}^{n-1}\lambda_{x_i}p_1(x_i|\lambda,\mu)\prod_{i=1}^{n}\frac{p_{n_i}(h|\lambda,\mu)}{p_1(h|\lambda,\mu)}$$

Proof. This follows directly from the approach taken in [2]. □

Corollary 4.

$$(1-p_0(t|\lambda,\mu)) = \frac{F'(t)}{\lambda_t F(t)}.$$

Proof. From Theorem 1 and Theorem 3 we obtain,

$$\lambda_t p_1(t|\lambda,\mu) = f(t).$$

This is equivalent to,

$$\lambda_t(1 - p_0(t|\lambda,\mu))\frac{1}{F(t)} = \frac{F'(t)}{F(t)^2},$$

which establishes the result. □

It remains an open question if Corollary 4 holds for all models specified in [1] with a coalescent point process.

References

[1] A. Lambert and T. Stadler. Birth–death models and coalescent point processes: the shape and probability of reconstructed phylogenies. *Theoretical Population Biology*, 90:113–128, 2013.

[2] T. Stadler and F. Bokma. Estimating speciation and extinction rates for phylogenies of higher taxa. *Systematic Biology*, 62(2):220–230, 2013.
