# Supplementary material for: Estimating shifts in diversification rates based on higher-level phylogenies
Source: Biol Lett. 2016 Oct;12(10):20160273. doi: 10.1098/rsbl.2016.0273 (PMC5095187; doi:10.1098/rsbl.2016.0273)
Supplement: Supplementary Figures [file rsbl20160273supp2.pdf]

Biology Letters

Estimating shifts in diversification rates based on higher-level phylogenies

Tanja Stadler & Jana Smrckova

Supplementary information: Figures

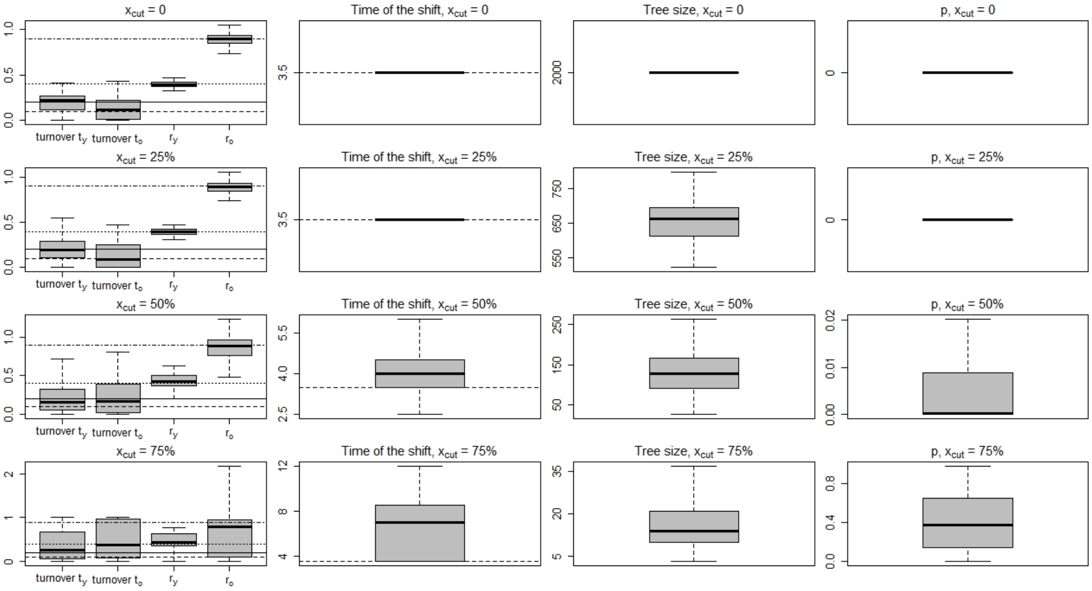

SUPPLEMENTARY FIGURE 1. Results of a simulation study for trees with constant extinction rate (0.1) and decreasing diversification rate (0.9 to 0.4) with a rate shift at 3.5 My before present.

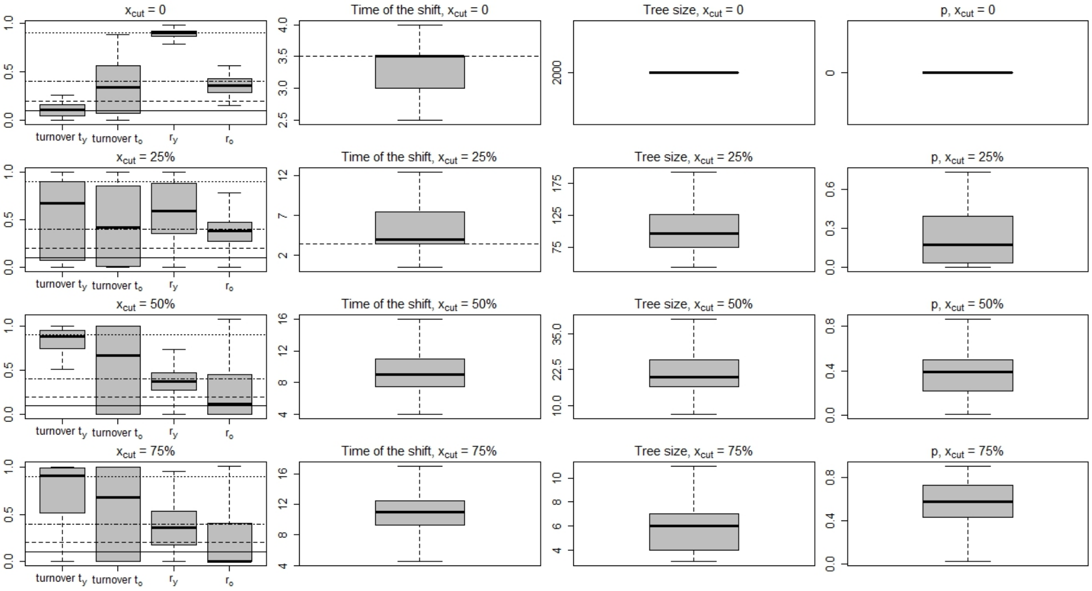

SUPPLEMENTARY FIGURE 2. Results of a simulation study for trees with constant extinction rate (0.1) and increasing diversification rate (0.4 to 0.9) with a rate shift at 3.5 My before present.

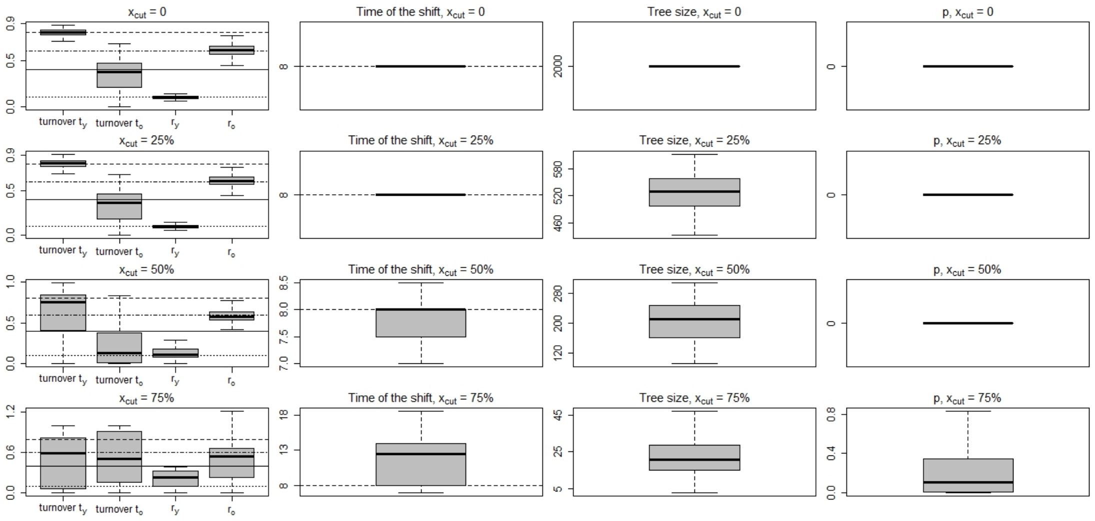

SUPPLEMENTARY FIGURE 3. Results of a simulation study for trees with constant extinction rate (0.4) and decreasing diversification rate (from 0.6 to 0.1) with a rate shift at 8 My before present.

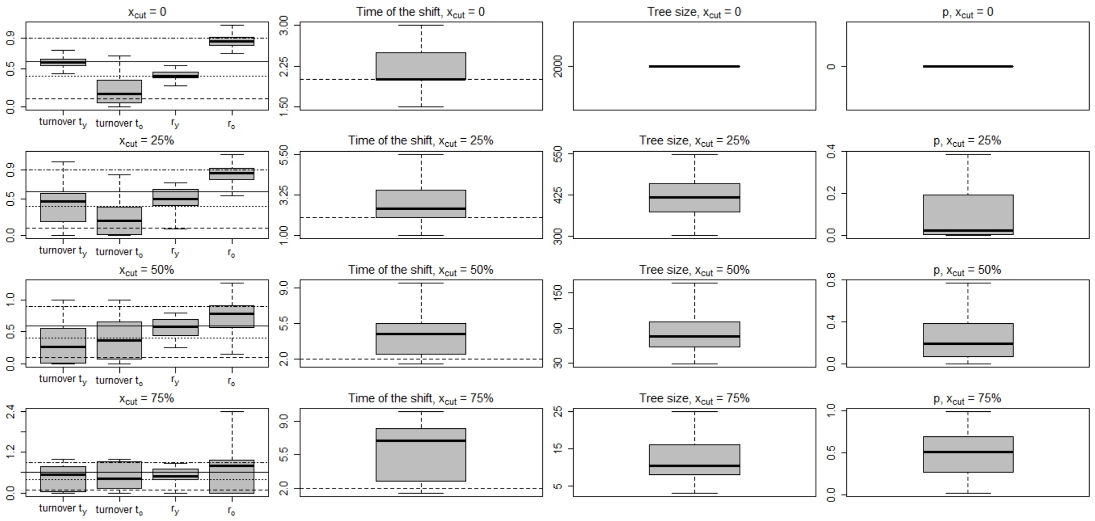

SUPPLEMENTARY FIGURE 4. Results of a simulation study for trees with constant speciation rate (1.0) and decreasing diversification rate (from 0.9 to 0.4) with a rate shift at 2 My before present.
